# Newer Anticoagulants for Non-Valvular Atrial Fibrillation

**DOI:** 10.3390/ph5050469

**Published:** 2012-05-04

**Authors:** Joseph M. Harburger, Wilbert S. Aronow

**Affiliations:** Department of Medicine, Cardiology Division, New York Medical College, Valhalla, NY 10595, USA

**Keywords:** atrial fibrillation, warfarin, anticoagulation, stroke, systemic embolism

## Abstract

Non-valvular atrial fibrillation is a recognized risk factor for stroke and systemic embolism. It has been clearly established that warfarin reduces the risk of stroke and systemic embolism in persons with atrial fibrillation and additional risk factors for stroke. The use of warfarin, however, requires frequent monitoring, and there is great variability in patient response to warfarin. Warfarin interacts with several medications and foods. In addition, warfarin use portends a significant risk of bleeding. For these reasons, warfarin is frequently not prescribed to persons for whom the drug would provide a clear benefit. Over the past decade, attempts have been made to develop drugs that are at least as safe and effective as warfarin for the treatment of atrial fibrillation that do not require monitoring nor have as many interactions. Initial studies of compounds in this regard ultimately failed due to safety concerns, but over the past two years two novel agents have been approved by the United States Food and Drug Association for anticoagulation in non-valvular atrial fibrillation, another drug is under review, and additional compounds are being studied. This article will review the use of warfarin and these new agents in the treatment of non-valvular atrial fibrillation.

## 1. Introduction

Non-valvular atrial fibrillation has long been known to be a risk factor for stroke and systemic embolism [1,2,3,4]. Atrial fibrillation has been shown to increase in prevalence with advancing age, and to be associated with stroke at all ages [2]. Data from over two decades ago revealed that therapy with either aspirin or warfarin is effective in decreasing the risk of stroke associated with atrial fibrillation [5]. In the Stroke Prevention Atrial Fibrillation (SPAF) study, therapy with either warfarin to maintain an international normalized ratio (INR) of 2.0–3.5 or aspirin 325 mg daily significantly decreased the risk of stroke over placebo in 1,244 patients with atrial fibrillation (stroke risk 1.6% per year with warfarin or aspirin, 8.3% per year with placebo, *p* < 0.00005). Later analysis of multiple studies revealed that warfarin was more efficacious in preventing stroke and systemic embolism than aspirin [6,7]. Subsequent studies such as the SPAF III trial identified patients with non-valvular atrial fibrillation who were at low risk for stroke on aspirin therapy [8].

Based on the SPAF III trial and other trials, risk stratification schemes were developed to assess individual patient risk of stroke with atrial fibrillation. Perhaps the most well known of these risk stratification schemes is the CHADS_2_ score, a scoring system for non-valvular atrial fibrillation to assess risk of stroke. To calculate a person’s CHADS_2_ score, a point is added for history of congestive heart failure, hypertension, age ≥75 years, and diabetes mellitus, and 2 points are added for history of stroke or transient ischemic attack. In an initial study involving 1,733 Medicare beneficiaries, the risk of stroke increased by a factor of 1.5 for each point increase in the CHADS_2_ score, from 1.9% per 100 patient years for a score of 0 to 18.2% for a score of 6 off of antithrombotic therapy [9]. More recent scoring schemes for risk of stroke in non-valvular atrial fibrillation have also been developed, such as the CHA_2_DS_2_-VASc score [10].

Scoring systems such as the CHADS_2_ score have largely impacted guidelines regarding the treatment of non-valvular atrial fibrillation such that recommendations regarding anticoagulation are made on individualized levels based on risk of stroke. Recent guidelines recommend that patients with a CHADS_2_ score of 0 receive no treatment with anticoagulants and those with a CHADS_2_ score of ≥1 without contraindications receive anticoagulation with warfarin to a goal INR of 2.0–3.0 [11,12].

While therapy with warfarin clearly decreases the stroke rate in patients with non-valvular atrial fibrillation, use of warfarin is fraught with many difficulties. Due to a marked variability of patient response to warfarin therapy and the interaction of warfarin with multiple foods and medications, achieving a therapeutic INR can be difficult and requires frequent blood draws for patient monitoring. Recent studies have shown that patients on warfarin frequently are not in the therapeutic range of anticoagulation [13,14]. In addition, the risk of major bleeding on warfarin is significant, particularly for patients ≥80 years and early in the course of therapy [15].

Warfarin therapy for patients with non-valvular atrial fibrillation has been used significantly less than recommended by guidelines, perhaps largely due to the difficulty in monitoring and risk of bleeding associated with this drug. [16,17,18,19]. With the advent of thienopyridines came hope that treatment with dual anti-platelet therapy could be as effective as warfarin therapy without the need for monitoring. However, studies have shown that though the addition of clopidogrel to aspirin reduces the risk of stroke in patients with non-valvular atrial fibrillation greater than aspirin alone, it increases the rate of bleeding and is inferior to warfarin in reducing stroke and systemic embolism [20,21]. Therefore, over the past several years new compounds have been developed with the goal of decreasing the risk of stroke and systemic embolism in atrial fibrillation without the inconveniences and risks associated with warfarin therapy. Below is a description of several of these compounds.

## 2. Ximelagatran

Ximelagatran was an oral direct thrombin inhibitor that required no laboratory monitoring to assess efficacy of anticoagulation. It was studied in the Stroke Prophylaxis using an ORal Thrombin Inhibitor in atrial Fibrillation (SPORTIF) III and SPORTIF V trials in a fixed dose of 36 mg twice daily *versus* adjusted dose warfarin to achieve an INR goal of 2.0–3.0 [22,23,24]. In these trials, ximelagatran was found to be non-inferior to warfarin in reducing the risk of stroke and systemic embolism, to cause similar rates of major bleeding, and to cause less overall bleeding. However, ximelagatran was shown to cause frequent elevations in liver enzymes in these trials, and was never approved for use in the United States due to concerns of hepatotoxicity.

## 3. Idraparinux

Idraparinux is a long-acting subcutaneous factor Xa inhibitor that was compared with warfarin in the comparison of idraparinux with vitamin K antagonists for prevention of atrial fibrillation (AMADEUS) trial in patients with non-valvular atrial fibrillation [25]. In this trial, patients with non-valvular atrial fibrillation at risk for stroke were randomized to either open-label warfarin with a goal INR of 2.0–3.0, or open label weekly subcutaneous idraparinux injections. This was a non-inferiority trial in which the primary efficacy endpoint was stroke or systemic embolism, and the primary safety endpoint was clinically significant bleeding. In this trial, 4,576 patients were randomized and patients were followed up for a mean of 10.7 months. Though idraparinux was determined to be non-inferior to warfarin in preventing stroke and systemic embolism, the trial was stopped early due to significantly increased bleeding with idraparinux. There was also an increased rate of intracranial bleeding with idraparinux. Based on the results of this study, idraparinux is no longer being considered as a treatment for non-valvular atrial fibrillation.

## 4. Dabigatran

Dabigatran is an oral direct thrombin inhibitor that requires no laboratory monitoring to assess efficacy of anticoagulation. Varying doses of the drug were first evaluated in patients with non-valvular atrial fibrillation in the dabigatran with or without concomitant aspirin compared with warfarin alone in patients with nonvalvular atrial fibrillation in the Prevention of Embolic and Thrombotic Events in Patients with Persistent AF (PETRO) study [26]. In this twelve week phase II trial, 502 patients with non-valvular atrial fibrillation were randomized in a blinded fashion to dabigatran in varying doses (50 mg twice a day, 150 mg twice a day, or 300 mg twice a day) plus or minus aspirin 81 mg or 325 mg daily, or randomized in an unblinded fashion to warfarin with a goal INR of 2.0–3.0. As the majority of dabigatran undergoes renal excretion, patients with creatinine clearance (CrCl) ≤30 mL/min were excluded from the study. The primary endpoints of the study were to evaluate the prevalence of stroke and systemic embolism and the risk of major bleeding in each group. This study revealed that major bleeding, defined as fatal or life-threatening bleeding, or bleeding requiring surgery, transfusion of ≥2 units of blood, or a drop in hemoglobin by ≥2.0 g/L, only occurred in patients taking dabigatran 300 mg twice a day plus aspirin. Stroke or systemic embolism only occurred in patients taking dabigatran 50 mg twice a day (plus or minus aspirin). No primary endpoints occurred in the groups receiving dabigatran 150 mg twice daily or warfarin during this brief trial period.

In a subsequent phase III multi-center study, dabigatran was compared to warfarin in non-valvular atrial fibrillation in the Randomized Evaluation of Long-Term Anticoagulation Therapy (RE-LY) trial [27,28]. In the RE-LY trial, 18,113 patients with non-valvular atrial fibrillation at risk of stroke were randomized in an unblinded fashion to either warfarin with a goal INR of 2.0–3.0, or dabigatran. Patients in the dabigatran group were then randomized in a blinded fashion to receive either 110 mg twice a day or 150 mg twice a day. Half of the patients enrolled in the trial were already receiving long-term warfarin therapy, and the mean CHADS_2_ score of patients in the trial was 2.1. Aspirin at a dose <100 mg or other antiplatelet agents were permitted at the discretion of the patients’ physicians. Patients with CrCl <30 mL/min were excluded, as were patients with recent stroke, high risk of bleeding, pregnancy, active liver disease, or valvular atrial fibrillation. The primary outcome was to assess whether dabigatran was non-inferior to warfarin in preventing stroke and systemic embolism. The primary safety outcome was to assess the risk of major hemorrhage on warfarin *versus* dabigatran defined as a drop in hemoglobin by ≥2.0 g/L, transfusion of at least two units of blood, or symptomatic bleeding in a critical area (including intracranial hemorrhage). The primary net clinical benefit was assessed via a composite of stroke, systemic embolism, pulmonary embolism, myocardial infarction, death, and major bleeding.

At a median follow-up of two years, dabigatran at a dose of 110 mg twice a day was shown to be non-inferior to warfarin in reduction of the primary outcome (stroke and systemic embolism) [28]. The primary endpoint occurred in 1.69% per year in the warfarin group and 1.53% in the dabigatran 110 mg group (*p* < 0.001 for non-inferiority). Dabigatran at a dose of 150 mg twice a day was shown to be superior to warfarin at reducing the primary endpoint (annual risk of primary endpoint was 1.1% in the 150 mg dabigatran group; *p* < 0.001 for superiority) [28]. The primary safety endpoint revealed that dabigatran at a dose of 110 mg twice a day caused less major bleeding than warfarin (risk of major bleeding 2.71% per year in the dabigatran group and 3.36% per year in the warfarin group; *p* = 0.003), and that there was no significant difference in the risk of major hemorrhage in the 150 mg dabigatran group and the warfarin group (risk of major bleeding 3.11% per year in the dabigatran group; *p* = 0.31). The risk of intracranial hemorrhage was significantly less in both doses of dabigatran as compared to warfarin (0.74% per year in the warfarin group compared to 0.23% per year in the 110 mg dabigatran group and 0.30% per year in the 150 mg dabigatran group). The net clinical outcome occurred in 7.64% of patients per year with warfarin *versus* 7.09% per year with 110 mg dabigatran (*p* = 0.10) and 6.91% of patients in the 150 mg dabigatran group (*p* = 0.04). There was a trend towards lower risk of death in the 150 mg dabigatran group *versus* the warfarin group, but this did not reach statistical significance (4.13% per year with warfarin *versus* 3.64% per year with 150 mg dabigatran, *p* = 0.051) [28]. The incidence of myocardial infarction was 0.53% per year for patients treated with warfarin *versus* 0.72% per year for patients treated with 110 mg of dabigatran (relative risk 1.35, *p* = 0.07) and 0.74% per year for patients treated with dabigatran 150 mg (relative risk = 1.38, *p* = 0.048).

Putting these data together, dabigatran at a dose of 110 mg twice a day was shown to be non-inferior to warfarin at preventing stroke and systemic embolism, and was less likely to cause major hemorrhage [28]. Dabigatran at a dose of 150 mg twice a day was superior to warfarin at preventing stroke and systemic embolism and did not cause any statistically significant difference in major bleeding [28]. Both doses of dabigatran were less likely than warfarin to cause intracranial bleeding.

Interestingly, dabigatran 150 mg twice a day increased the risk of gastrointestinal bleeding as compared to warfarin and dabigatran 110 mg twice a day. Though the mechanism of this is unclear, the authors hypothesized that the reason for this was that the acidic content of dabigatran tablets necessary for their absorption promoted gastrointestinal bleeding. In addition, the most common adverse side effect of dabigatran was dyspepsia, and the authors believed that this was also related to the acid content of the tablets. Unlike in the previous trials evaluating ximelagatran, another oral direct thrombin inhibitor, rates of hepatotoxicity were not increased in the dabigatran groups compared to the warfarin group [22,23,24].

A subgroup analysis of the RE-LY trial was done to compare the relative benefit of dabigatran as compared to warfarin for varying CHADS_2_ scores [29]. The risk of stroke or systemic embolism and the risk of major and intracranial bleeding increased as CHADS_2_ score increased (groups analyzed were those with CHADS_2_ scores of 0, 1, 2, and 3–6). Results comparing dabigatran to warfarin in the RE-LY trial were consistent across groups with different CHADS_2_ scores, suggesting that the RE-LY study results could be applied to patients with varying risk of stroke as per CHADS_2_ risk assessment.

Additional analysis was done to determine if the RE-LY study results were largely due to varying degrees of INR control at different study sites, with the thought that event rates would be higher in patients on warfarin with INR values outside of the therapeutic range of 2.0–3.0 [30]. Analysis revealed that dabigatran 110 mg twice a day resulted in major bleeding less often compared to warfarin regardless of INR control, while patients with the most optimal INR control on warfarin had similar major bleeding rates to those on dabigatran 150 mg twice daily. Patients taking either dabagitran dose were less likely to have stroke or systemic embolism when the INR was poorly controlled on warfarin, while there were similar event rates when the INR was in the therapeutic range.

Based on the results of the RE-LY trial, dabigatran was approved for use in non-valvular atrial fibrillation by the United States Food and Drug Administration in October 2010 [31]. In the United States, a dose of 150 mg twice a day is approved for patients with CrCl >30mL/minute. In addition, based on pharmacokinetic analysis, dabigatran at a dose of 75mg twice daily was approved in patients with CrCl of 15–30 mL/min, while dabigatran is not approved for persons with a CrCl <15 mL/min.

## 5. Rivaroxaban

Rivaroxaban is an oral factor Xa inhibitor that provides anticoagulation without the need for monitoring that was recently studied for use in non-valvular atrial fibrillation in the Rivaroxaban Once Daily Oral Direct Factor Xa Inhibition Compared With Vitamin K Antagonism for Prevention of Stroke and Embolism Trial in Atrial Fibrillation (ROCKET AF) [32,33]. This trial was a double-blind, double-dummy multi-center randomized control trial. The trial evaluated rivaroxaban once daily *versus* warfarin to maintain INR of 2.0–3.0 in 14,264 patients with non-valvular atrial fibrillation at moderate to high risk for stroke (CHADS_2_ score of 2 or greater). Patients were given both a medication (either warfarin or rivaroxaban) and a placebo tablet to maintain blinding. Patients taking warfarin were provided with INR values prompting adjustment of warfarin dosing, if necessary, and patients taking rivaroxaban were provided with “sham” INR values prompting “dosing adjustments” of the placebo tablets if needed. Patients with CrCl >50 mL/min on rivaroxaban were given a dose of 20 mg daily, while those with CrCl of 30–49 mL/min were given a dose of 15 mg daily; patients with CrCl of <30 mL/min were excluded from the trial. Ten percent of the patients enrolled in the trial had a CHADS_2_ score of 2 without prior stroke, transient ischemic attack or embolism, and the remainder of patients either had prior embolism or a CHADS_2_ score of 3 or greater. The mean CHADS_2_ score of all patients enrolled was 3.5 and the median score was 3.0. Primary analysis was done to determine if rivaroxaban was non-inferior to warfarin in preventing stroke or systemic embolism, and testing for superiority was performed when non-inferiority was established. Primary safety analysis was performed to determine the rate of major and clinically relevant non-major bleeding in the rivaroxaban and warfarin groups. Secondary analysis was a composite of stroke, systemic embolism, myocardial infarction, and death from cardiovascular causes, and each component evaluated independently.

Patients were treated for a median of 590 days in the study. In intention-to-treat analysis, stroke or systemic embolism occurred in 2.1% per year in the rivaroxaban group and 2.4% per year in the warfarin group, establishing non-inferiority for rivaroxaban (*p* < 0.001) with a trend toward superiority that did not meet statistical significance (*p* = 0.12) [33]. There was no statistically significant difference in major or non-major clinically relevant bleeding in the rivaroxaban group (14.9% per year) *versus* the warfarin group (14.5% per year), however, rivaroxaban was associated with lower rates of intracranial and fatal bleeding that met statistical significance. Secondary analyses using the endpoints described above did not reveal statistically significant differences in outcomes. Of note, patients randomized to rivaroxaban were more likely to have gastrointestinal bleeding, similar to what was seen in the RE-LY trial comparing dabigatran and warfarin described above.

Largely as a result of this trial, the United States Food and Drug Administration approved rivaroxaban for the prevention of stroke and systemic embolism in non-valvular atrial fibrillation [33]. A dose of 15 mg once daily is recommended for patients with a CrCl of 15–50 mL/min and a dose of 20 mg once daily is recommended for patients with a CrCl of >50 mL per minute. Like dabigatran, rivaroxaban is not approved for use in persons with a CrCl <15 mL per minute.

## 6. Apixaban

Apixaban, like rivaroxaban, is an oral factor Xa inhibitor that provides anticoagulation without the need for monitoring. Apixaban was studied in the Apixaban *Versus* Acetylsalicylic Acid (ASA) to Reduce the Risk of Stroke in Atrial Fibrillation Patients Who Have Faileor Are Unsuitable for Vitamin K Antagonist Treatment (AVERROES) trial in patients with non-valvular atrial fibrillation unable or averse to taking warfarin, where it was compared to aspirin therapy in patients with peripheral arterial disease and/or a CHADS_2_ score ≥1 [34]. In this trial, 5,599 patents with a mean CHADS_2_ score of 2.1 were randomized in a double-blind manner to receive either apixiban 5 mg twice daily or aspirin 81 to 324 mg daily. The primary outcome was the prevalence of stroke or systemic embolism. There were significantly less strokes and systemic embolic events in the apixaban *versus* aspirin group (1.6% per year *versus* 3.7% per year; *p* < 0.001) without a statistically significant difference in major or intracranial bleeding between groups. The results favored apixaban to such a degree that the data and safety monitoring board for the trial terminated the study early.

Following AVERROES, apixaban was compared to warfarin with a goal INR of 2.0–3.0 in the Apixaban for Reduction in Stroke and Other Thromboembolic Events in Atrial Fibrillation (ARISTOTLE) trial [35]. In this trial, apixaban was compared to warfarin in 18,201 patients with non-valvular atrial fibrillation and a CHADS_2_ score ≥1 (mean CHADS_2_ score = 2.1). A double-blind, double-dummy trial design was used, in which patients were given both a medication (either warfarin or apixaban) and a placebo tablet to maintain blinding. The majority of patients (n = 17,730) were randomized to warfarin or apixaban 5 mg twice a day, while a small subgroup of patients with two or more criteria thought to increase bleeding risk on apixaban (age ≥80 years, weight ≤60 kg, CrCL 1.5 mg/dL) were randomized to warfarin or apixaban 2.5 mg twice daily. Patients taking warfarin were provided with INR values prompting adjustment of warfarin dosing, if necessary, and patients taking apixaban were provided with “sham” INR values prompting “dosing adjustment” of the placebo tablets if needed. Patients were followed for a median of 1.8 years in the study. The primary analysis was to establish whether apixaban is non-inferior to warfarin in preventing stroke and systemic embolism. The primary safety analysis was to determine the risk of major bleeding associated with each drug. Secondary analyses were performed to determine whether apixaban is superior to warfarin at reducing the primary endpoint, and to determine whether apixaban was associated with a difference in the risk of death from any cause. Patients were excluded from the trial if they had valvular atrial fibrillation, conditions other than atrial fibrillation requiring anticoagulation, stroke within one week, need for aspirin >165 mg per day or need for dual antiplatelet therapy with aspirin and clopidogrel, or CrCl <25 mL per minute or serum creatinine >2.5 mg/dL. The median age of patients in the trial was 70 years old, and 57% of patients were on prior warfarin therapy.

The study results strongly favored the use of apixaban in the study population. The primary outcome was 1.27% per year in the apixaban group *versus* 1.60% per year in the warfarin group, a statistically significant finding demonstrating both non-inferiority and superiority for apixaban as compared to warfarin (*p* = 0.01 for superiority) [35]. Apixaban was found to be statistically superior to warfarin in reducing the risk of major bleeding, hemorrhagic stroke, and death from any cause. Unlike dabigatran in the RE-LY trial and rivaroxaban in the ROCKET AF trial, gastrointestinal bleeding was not more common in the apixaban group than the warfarin group. Findings in the small subgroup of patients who were randomized to the lower apixaban dose *versus* warfarin due to presumed increased bleeding risk were similar to those in the group randomized to apixaban 5 mg twice daily *versus* warfarin.

The United States Food and Drug Administration accepted a New Drug Application for review of apixaban in the prevention of stroke and systemic embolism in non-valvular atrial fibrillation in November 2011 [36]. At the time of this writing, the drug is still under review.

## 7. Other Agents

Other agents are currently being investigated for prophylaxis against stroke and systemic embolism in non-valvular atrial fibrillation. Edoxaban is an oral factor Xa inhibitor that was compared with warfarin in a 12 week phase II safety trial [37]. In this study of 1,146 patients with non-valvular atrial fibrillation, edoxaban at doses of 30 mg daily and 60 mg daily was found to cause similar rates of bleeding and liver function abnormalities as warfarin. A randomized, double blind, double dummy phase III study named Effective aNticoagulation with factor Xa next GEneration in Atrial fibrillation-Thrombolysis in Myocardial infarction study 48 (ENGAGE AF-TIMI 48) is currently ongoing to compare the efficacy and safety of edoxaban with warfarin in non-valvular atrial fibrillation [38,39,40,41,42,43].

Betrixaban, an oral factor Xa inhibitor, was compared to warfarin in patients with non-valvular atrial fibrillation with an additional risk factor for stroke in the Randomized Clinical trial of Three Doses of a Long-acting Oral direct Factor Xa Inhibitor Betrixaban in Patients With Atrial fibrillation (EXPLORE-Xa) trial [38,42,43]. In this trial, betrixaban at a dose of 40 mg daily was shown to cause less bleeding than warfarin at a goal INR 2.0–3.0, and betrixaban at doses of 60 mg daily and 80 mg daily were shown to cause similar rates of bleeding. No hepatotoxicity was seen. Betrixaban has a half-life that would presumably allow for once a day dosing, is cleared solely in the bile and thus presumably could be used in all degrees of renal failure, and is reportedly being developed with an antidote that would make this drug reversible in the case of bleeding or need for emergent surgery. Betrixaban at doses of 60 mg and 80 mg daily caused more diarrhea, constipation, and nausea than warfarin [38,43]. Additional studies are needed to determine if there is a role for betrixaban in non-valvular atrial fibrillation.

Concerns about the use of the newer anticoagulants include the absence of a monitoring test to evaluate the status of patients receiving oral direct thrombin inhibitors or factor Xa inhibitors. There are also no available antidotes to treat patients receiving direct thrombin inhibitors of factor Xa inhibitors in emergency situations.Patients on these medications should avoid concomitant use of drugs that increase bleeding including glycoprotein IIb/IIIa inhibitors and nonsteroidal antiinflammatory drugs. There is no head-to-head comparison of these newer anticoagulants with each other. It remains to be seen how safe and effective these new anticoagulants are in clinical practice. Compliance might be a problem with drugs administered twice daily such as dabigatran or apixaban. Experience with cardioversion is also limited with these newer anticoagulants, and no guidelines encourage their use at the time of pharmacological or direct current cardioversion.

## 8. Conclusions

Atrial fibrillation is a major cause of stroke and systemic embolism, and warfarin has been shown to be an effective agent at preventing these potentially devastating consequences. Because of the many difficulties associated with taking warfarin, however, many patients are not prescribed warfarin despite this benefit. In addition, persons taking warfarin have the burden of dealing with drug and dietary interactions and frequent blood draws. Even with optimal management of warfarin, persons frequently have INR values that fall out of the therapeutic range, subjecting them to a greater risk of stroke and systemic embolism when the INR is subtherapeutic, and of bleeding when the INR is supratherapeutic. For the past decade, investigations have focused on new compounds to prevent stroke and systemic embolism in atrial fibrillation that do not carry the multiple burdens associated with warfarin therapy. Over the past couple of years, three of these compounds have been shown to be at least as safe and effective as warfarin the prevention of stroke and systemic embolism in non-valvular atrial fibrillation, and two of these drugs are now on the market in the United States. While these new medications are more expensive than warfarin, several recent studies have shown that their use is cost-effective [44,45,46,47]. Furthermore, additional compounds are in the process of being studied. Over the next few years, these new agents will likely supplant the use of warfarin in the large majority of patients with non-valvular atrial fibrillation.
